# Clinical study of ^99m^Tc-3P-RGD2 peptide imaging in osteolytic bone metastasis

**DOI:** 10.18632/oncotarget.17486

**Published:** 2017-04-27

**Authors:** Guoqiang Shao, Wei Gu, Muhong Guo, Shiming Zang, Jinjing Fu, Shuang Liu, Feng Wang, Zizheng Wang

**Affiliations:** ^1^ Department of Nuclear Medicine, Nanjing First Hospital, Nanjing Medical University, Nanjing, China; ^2^ Department of Respiration, Nanjing First Hospital, Nanjing Medical University, Nanjing, China; ^3^ School of Health Sciences, Purdue University, West Lafayette, IN, USA

**Keywords:** osteolytic metastasis, targeted imaging, integrin αvβ3, RGD peptide, lung cancer

## Abstract

**Objective:**

To investigate the value of integrin α_v_β_3_ targeted imaging with ^99m^Tc-HYNIC-PEG_4_-E[PEG_4_-c(RGDfk)]_2_ (^99m^Tc-3P-RGD_2_) as a radiotracer in dectecting osteolytic bone metastases.

**Methods:**

This is a retrospective study involving a cohort of 69 consecutive patients including 59 with lung cancer and 10 with other cancers. Patients were required to receive whole body scan (WBS) and regional SPECT/CT imaging with ^99m^Tc-3P-RGD_2_ (RGD imaging) and ^99m^Tc-MDP (MDP imaging) as a radiotracer successively within days. Final diagnosis was based on comprehensive assessment of all available data including case history, CT, MRI, SPECT/CT, PET/CT, histopathology and 6-12 months follow-up. Visual observation and semiquantitative analysis (T/N: tracer uptake ratio of osteolytic metastases to normal bone) of ^99m^Tc-3P-RGD_2_ or ^99m^Tc-MDP imaging were performed and their detective values for osteolytic metastases were compared.

**Results:**

A total of 131 osteolytic metastatic lesions were retrospectively studied. Osteolytic metastases mainly presented as “hot region”, occasionally as “cool or normal region” on RGD imaging. The detection sensitivity of RGD WBS for osteolytic metastases was significantly higher than that of ^99m^Tc-MDP WBS (80.9% vs. 46.6%, *p*<0.01). The sensitivity increased to 96.2% (126/131) when combining with SPECT/CT. ^99m^Tc-3P-RGD_2_ imaging also promoted the detection of unknown primary tumor, lymph node metastases and offered information for clinical staging. T/N of ^99m^Tc-3P-RGD_2_ in lung adenocarcinoma osteolytic metastases showed no statistical difference compared with that in squamous-cell carcinoma (6.84±3.46 *vs*. 7.33±3.22, t = 0.39, *p* = 0.71). Whereas, it was higher in osteolytic metastases from lung cancer than that from thyroid cancer (7.05±3.01 *vs*. 4.11±2.67, *p* = 0.03).

**Conclusion:**

^99m^Tc-3P-RGD_2_ peptide imaging showed great potential for detection of osteolytic bone metastasis due to high expression level of integrin αvβ3 on osteoclast and most tumor cells.

## INTRODUCTION

Skeleton is one of the most common sites for metastasis. A majority of patients may present osteolytic and osteoblastic metastasis, which will result in marked disturbances of bone remodeling that can be lytic and/or blastic. Osteolytic metastasis, a common complication in breast cancer, lung cancer, prostate cancer, or multiple myeloma [1, 2], brings about significant morbidity of intractable pain, spinal cord compression, pathologic fracture, functional impairment and hypercalcemia [3, 4]. In addition, bone micro-metastasis can serve as an independent predictor of poor outcome in patients with tumor even among lymph node-negative patients with primary tumors of less than 2 cm [5]. Early diagnosis of osteolytic metastasis is definitely important for its correct treatment and better prognosis.

Anatomical imaging (e.g. CT and MRI) has been commonly used for the diagnosis of osteolytic lesions. However, the diagnostic sensitivity of CT is relatively low in cases of a mineral content loss of less than 50% at the lesions sites [6]. Meanwhile, MRI could not readily display bone degradation [7]. ^99m^Tc-diphosphonates (^99m^Tc-MDP)-based planar bone scan is considered as the standard technique for the detection of skeletal metastasis as it shows high sensitivity, especially for bone lesions with osteogenesis. As is known to all, the accumulation of ^99m^Tc-MDP in the bone is highly relied on the osteoblast activity [8]. Absence of ^99m^Tc-MDP or high false negative was reported at early period in osteolytic lesions rich in osteoclast, tumor cells and different degree of osteolysis. Therefore, the detection value of ^99m^Tc-MDP imaging in osteolytic lesions was lower than that in the osteoblastic lesions [9]. On this basis, it is urgent to develop appropriate imaging technique(s) for the detection of osteoclasts and adjacent tumor cells in patients with osteolytic metastases in an early and accurate manner.

Malignant interaction between tumor cells and osteoclasts in bone microenvironment plays a pivotal role in the pathogenesis of metastatic bone disease such as osteolytic and/or osteoblastic bone metastases. Tumor cells contributed to the recruitment and activation of osteoclasts, which triggered regional osteolysis and tumor cell proliferation [10]. Tumor cells and osteoclasts are the main component of osteolytic metastatic lesions. Integrin α_v_β_3_, highly expressed in several tumor cells and activated endothelial cells in newly-generated vessels, has been considered as a target for tumor imaging with radiolabeled arginine-glycine-aspartic acid (RGD) peptides and analogues. Furthermore, osteoclast expressing high level of integrin α_v_β_3_ had attracted great attention for positive imaging of osteolytic bone metastasis [11].

^99m^Tc-HYNIC-3PEG4-E[c(RGDfK)2] (^99m^Tc-3P-RGD_2_) is a cyclic RGD dimmer peptide with high specificity and affinity to integrin α_v_β_3_. To date, ^99m^Tc-3P4-RGD_2_ scintigraphy has been commonly used for differential diagnosis of solitary pulmonary nodule, lymph node metastasis and treatment response monitoring [12-15]. This retrospective study was designed to investigate the detective and diagnostic value of ^99m^Tc-3P4-RGD_2_ imaging for osteolytic bone metastasis.

## MATERIALS AND METHODS

### Patients

This study was approved by the Institute Review Boards of both Nanjing Medical University and Nanjing First Hospital. Written informed consent was obtained from each patient. All reported investigations were conducted in accordance with the Declaration of Helsinki and with our national regulations. Eighty-eight patients were diagnosed with malignant tumors such as primary lung cancer, lung metastases from thyroid cancer, malignant chromaffin-cell tumor, gastric cancer, breast cancer based on the assessment of case history, CT, MRI, SPECT/CT, PET/CT histopathology and 6-12 month follow-up data. Sixty-nine (48.6%) patients presenting pulmonary nodule were suspected with lung cancer and concurrent osteolytic bone metastases. The exclusion criteria were as follows: (i) received treatment before imaging; (ii) pregnant and breastfeeding patients; (iii) those with a history of bone trauma, fracture, and bone inflammation such as tuberculosis within one year; or (iv) those could not accomplish the required examinations because of severe pain or claustrophobia.

### Radiopharmaceutical preparation

^99m^Tc-3P-RGD_2_ was prepared according to our previous description[16, 17]. Briefly, 1 mL Na^99m^TcO4 solution (1, 110-1, 850 MBq) was added into lyophilized kit formulation containing 20 mg hydrazinonicotinamide-PEG4-E[PEG4-c(RGDfK)]_2_ (HYNIC-3P-RGD_2_), 6.5 mg tricine, 5 mg trisodium triphenylphosphine-3, 39, 399-trisulfonate (TPPTS), 38.5 mg disodium succinate hexahydrate, 40 mg mannitol, and 12.7 mg succinic acid. The reaction system was incubated in water at 100°C for 20 min. The radiochemical purity of the product was > 98% by radio-HPLC. Radiopharmaceuticals were then subject to a 0.20mm Milex-LG filter and diluted to 740MBq/ml for clinical study.

### Imaging protocol

We evaluated all of the patients using ^99m^Tc-3P-RGD_2_ imaging and ^99m^Tc-MDP imaging [including both whole body scan (WBS) and SPECT-CT with consistent imaging field] within one week. The imaging was performed on a SPECT-CT equipment (Symbia T6, Simense, Germany) according to manufacturer’s instructions. All patients had urinated completely prior to imaging. Imaging was performed within one week. Anterior and posterior WBS was performed 1h after intravenous injection of ^99m^Tc-3P-RGD_2_ (750±37.5MBq, in 1.0 ml saline) or 3h after intravenous injection of ^99m^Tc-MDP (750±75MBq, in 1.0 ml saline). WBS imaging parameters included low-energy high-resolution collimators, bed movement speed of 10.0 cm/min, energy window of 20% width and centered on 140keV. SPECT data set was obtained (ZOOM of 1.3, 256 by 256 matrix size, 30s/Frame for 32 frames and 180° each head of the dual head-camera) immediately after WBS using a low-energy high-resolution collimator at 140 keV with a window width of 20%. Then the data were reconstructed with ordered subset expectation maximization (OSEM). CT scan was performed in the same anatomic locations as SPECT with a tube voltage of 130 kV and current intensity at 120 mA/slice with a slice thickness of 3 mm.

### Image analysis

Image analysis was performed by 2 nuclear medicine physicians and 2 radiologists blinded to the case history, examination results, and pathologic diagnosis or follow up data. Visual observation and semiquantitative analysis of ^99m^Tc-3P-RGD_2_ or ^99m^Tc-MDP imaging were performed based on the accumulation of radiotracer at the lesion sites comparing to the contralateral or surrounding normal bone tissues (T/N ratio). The 4-point grade system was also adopted to describe the uptake degree of radiotracers in osteolytic lesions [18]: grade 0, tracer uptake similar to surrounding normal bone structure; grade 1, uptake less (^99m^Tc-MDP) or slightly higher (^99m^Tc-3P-RGD_2_) than surrounding normal bone structure; grade 2, uptake significantly less (^99m^Tc-MDP) or higher (^99m^Tc-3P-RGD_2_) than surrounding normal bone structure; grade 3, abnormal aggregation of ^99m^Tc-3P-RGD_2_ or almost absence of ^99m^Tc-MDP with or without regional accumulation surrounding the lesion or regional aggregation of ^99m^Tc-MDP in confirmed osteolytic lesions. Osteolytic bone metastasis was ascertained according to any of the following criteria: tracer uptake score greater or equal to grade 2 on either ^99m^Tc-3P-RGD_2_ or ^99m^Tc-MDP imaging; tracer uptake score equals to grade 1 on both ^99m^Tc-3P-RGD_2_ and ^99m^Tc-MDP imaging especially with bone pain symptom, or increased uptake on ^99m^Tc-MDP imaging but confirmed to be osteolytic on other imaging modalities such as CT (including CT on SPECT-CT imaging), ^18^F-FDG PET-CT imaging, and MRI.

### Verification of osteolytic bone metastases

The final diagnosis of osteolystic bone metastases was based on histopathology, imaging findings (i.e. CT, SPECT/CT, PET/CT, MRI) and clinical follow-up data using specific standards as follows: (a) histopathologically proven; and (b) Besides primary malignancy diagnosis, CT or magnetic resonance imaging (MRI) results indicated obvious bone destruction without osteogenic imaging performance. (c) Increased range and/or lesions of bone destruction during follow-up [19-21]. During the clinical follow-up, multiple imagings were required for the patients, and all patients were followed up at least 6 months. Lesions exhibiting both osteolytic and osteosclerotic changes were verified by either type, depending on the predominant changes [22].

### Statistical analysis

Data were expressed as mean ± standard deviation. The number of osteolytic bone metastases detected using ^99m^Tc-3P-RGD_2_ and ^99m^Tc-MDP was compared. Sensitivity of ^99m^Tc-3P-RGD_2_ imaging and ^99m^Tc-MDP imaging modality were compared using McNemar test. The lesion detection consistency of the two modalities were compared using the Kappa test. *P* < 0.05 was considered to be statistically significant.

## RESULTS

### Patient characteristics and distribution of osteolytic lesions

Sixty-nine cases with malignant tumor were confirmed with 131 osteolytic metastases according to the comprehensive data based on case history, CT, MRI, SPECT/CT, PET/CT examinations, histopathology and 6-12 monthes follow-up. Patients’ characteristics and the number of osteolytic, lymph node metastases were listed in Table 1.

**Table 1:** Demographic profile and the final histological diagnosis of 69 patients with malignant tumors and distant metastasis

Category	number of people	proportion (%)	number of osteolytic metastasis	number of lymph node metastasis
**Gender**				
Male	39	56.5		
Female	30	43.5		
**Tumor type**				
Lung cancer				
adenocarcinoma	27	39.1	74	4
Squamous-cell carcinoma	32	46.4	40	15
Thyroid cancer	3	4.3	4	2
Malignant chromaffin-cell tumor	2	2.9	5	
breast cancer	1	1.4	1	0
Gastric cancer	4	5.8	5	1

### Visual analysis of osteolytic bone metastasis on ^99m^Tc-MDP and ^99m^Tc-3P-RGD_2_ imaging

The distribution of the 131 osteolytic bone metastases was summarized in Table 2. Among these metastases, 57 were localized in the neck and chest including cervical vertebra (8), thoracic vertebra (11), sternum (7), rib (31), 20 in the lumbar vertebra, 33 in the sacrococcyx and pelvis, and 21 in the appendicular skeleton including limb (14) and shoulder (7).

**Table 2:** Comparative analysis of ^99m^Tc-3P-RGD_2_ and ^99m^Tc-MDP whole body scan imaging (WBS) in the detection of osteolytic metastases in axial, appendicular skeleton

Bone area	number of lesions	WBS		*p* value
		^99m^Tc-MDP	^99m^Tc-3P-RGD_2_	
axial skeleton	110	60	92	<0.001
appendicular skeleton	21	17	19	>0.05
total	131	77	111	<0.001

**Figure 1:**
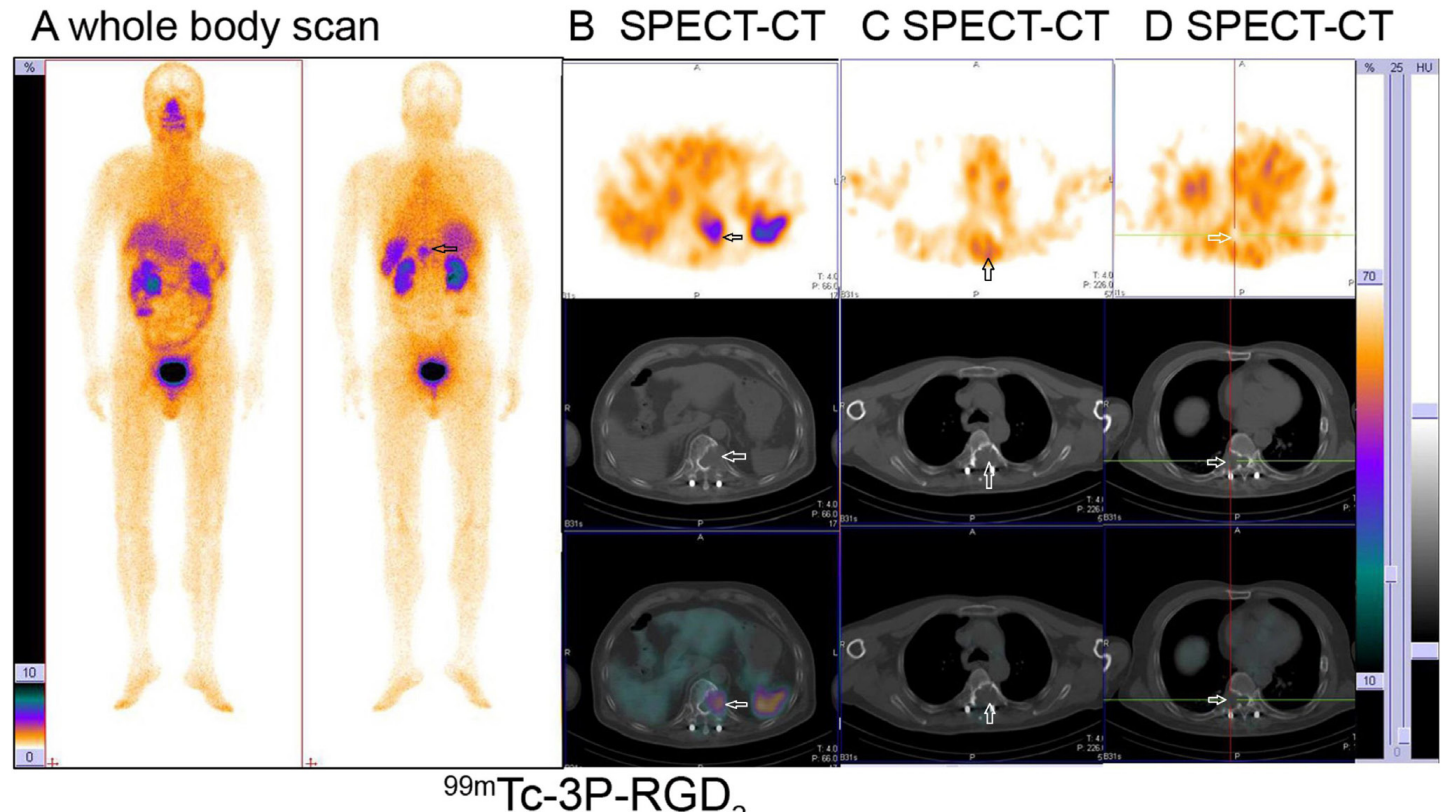
Osteolytic bone metastases from lung cancer displayed as significant accumulation (hot area) (A, B), slight higher uptake (C) or absence (cold area) (D) of ^99m^Tc-3P-RGD_2_ on whole body planar scan (A) and SPECT-CT (B, C, D) with bone destruction on CT (see arrows)

**Figure 2:**
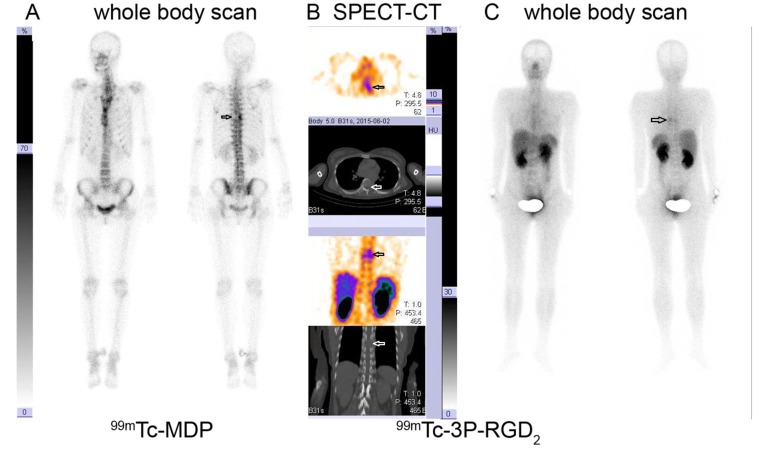
^99m^Tc-MDP Whole Body Scan (WBS) *versus* ^99m^Tc-3P-RGD_2_ WBS and SPECT-CT in a lung cancer patient ^99m^Tc-MDP WBS demonstrated tracer absence (cold area) in the left side of the 7^th^ thoracic vertebral body **A.** ^99m^Tc-3P-RGD_2_ SPECT-CT imaging demonstrates bone destruction and corresponding tracer accumulation in the left side of 7^th^ thoracic vertebral body and adjacent rib with T/N ratio of 6.31 **B.** ^99m^Tc-3P-RGD2 WBS displayed as “hot region” in the left side of the 7^th^ thoracic vertebral body **C.** A: ^99m^Tc-MDP WBS; B: ^99m^Tc-3P-RGD_2_ SPECT-CT; C: ^99m^Tc-3P-RGD_2_ WBS. (see arrows)

**Figure 3:**
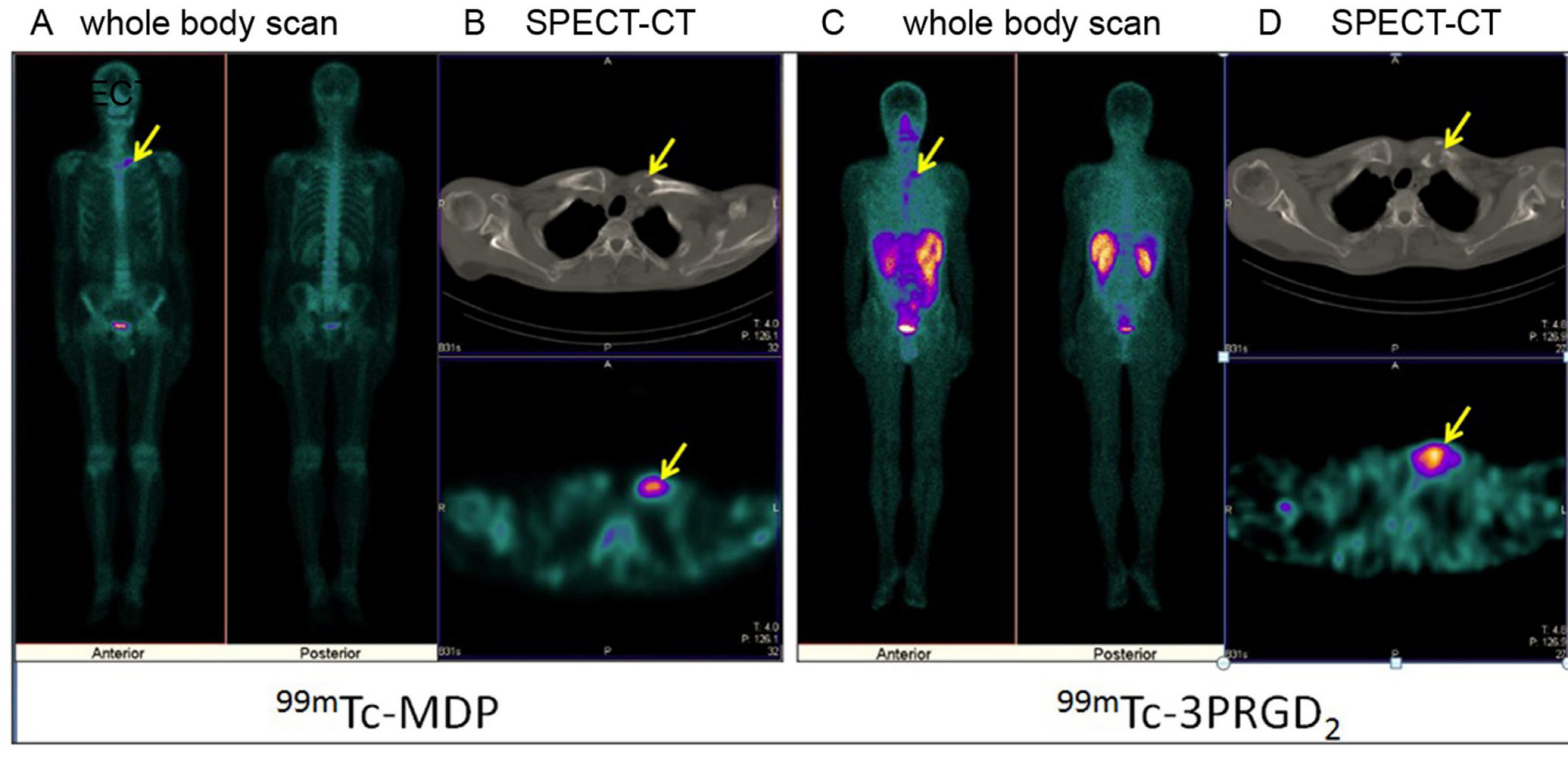
^99m^Tc-MDP imaging versus ^99m^Tc-3P-RGD_2_ imaging [Whole Body Scan (WBS) and SPECT-CT) in a patient with lung cancer Osteolytic bone metastases in the left clavicular head demonstrates as “hot region” on both ^99m^Tc-MDP **A.**, **B.** and ^99m^Tc-3P-RGD_2_ imaging **C.**, **D.** and bone destruction on CT(B,D) . A: ^99m^Tc-MDP WBS; B: ^99m^Tc-MDP SPECT-CT; C: ^99m^Tc-3P-RGD_2_ WBS; B: ^99m^Tc-3P-RGD_2_ SPECT-CT. (see arrows).

Based on visual analysis, osteolytic bone metastases were mainly manifested as significant accumulation (hot area, Fig. 1A and 1B), occasionally slight higher uptake (Fig. 1C) or absence of ^99m^Tc-3P-RGD_2_ on WBS and SPECT-CT imaging with or without bone destruction on CT (cold area, Fig. 1D). For ^99m^Tc-MDP, osteolytic bone metastases were manifested as “cold area” and occasionally as increased uptake (Fig. 2 and 3). Single osteolytic metastasis (with a long diameter of 4.1 cm) was manifested as cold region with slight elevation of ^99m^Tc-MDP uptake in the peripheral part, while negative findings were observed on ^99m^Tc-3P-RGD_2_ WBS for the single osteolytic metastasis.

When scored visually based on whole body scan, 30.5% (40/131) of osteolytic metastatic lesions was graded equally between ^99m^Tc-MDP and ^99m^Tc-3P-RGD_2_, 57.3% (75/131) was graded higher on ^99m^Tc-3P-RGD_2_ and 12.2% (16/131) was graded higher on ^99m^Tc-MDP. Comparative analysis of ^99m^Tc-3P-RGD_2_ and ^99m^Tc-MDP whole body scan imaging (WBS) in the detection of osteolytic metastases was summarized in Table 2. The sensitivity of ^99m^Tc-3P-RGD_2_ WBS on metastatic lesions was superior to that of ^99m^Tc-MDP WBS, especially in axial skeleton.

**Figure 4:**
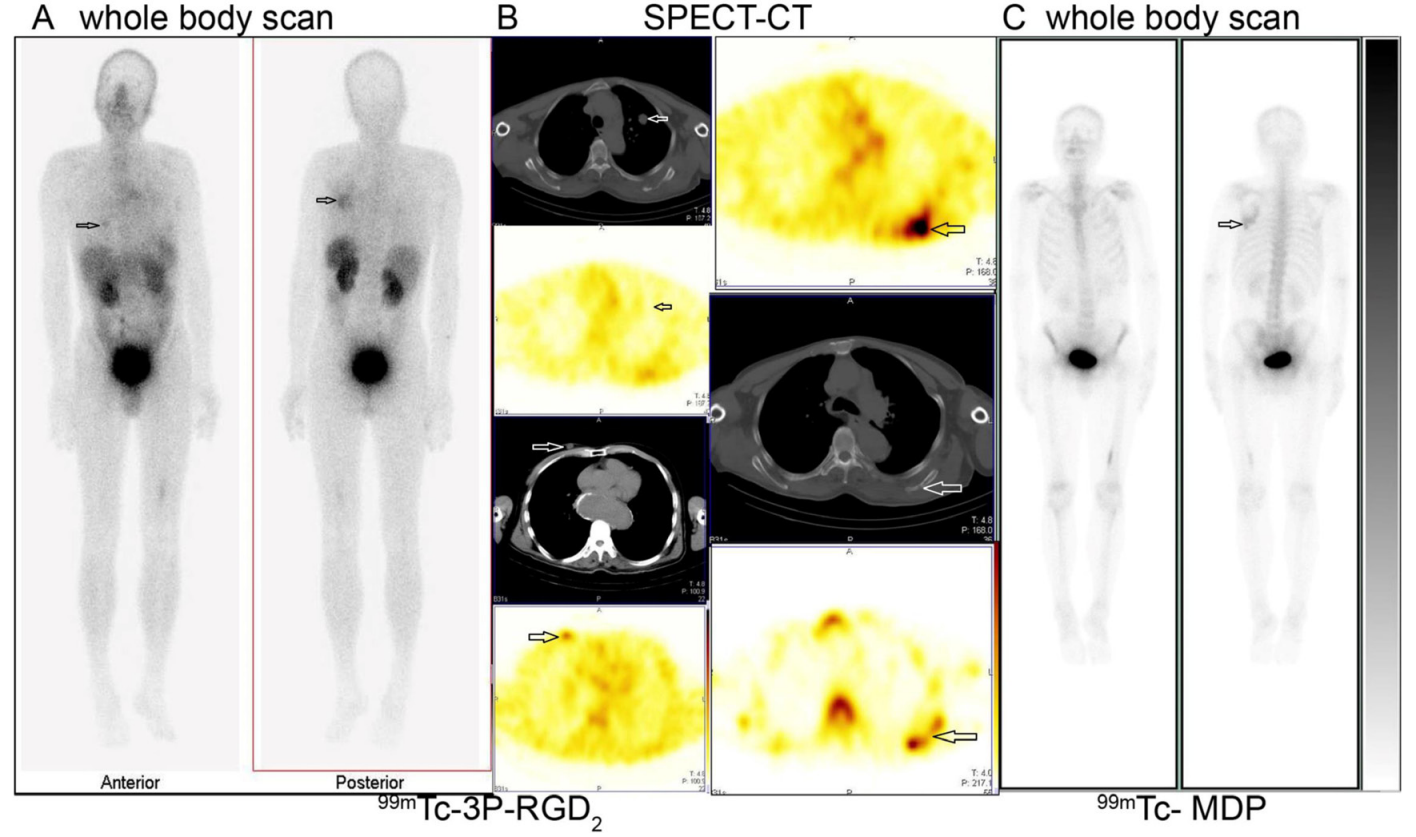
^99m^Tc-3P-RGD_2_ imaging promoted the detection of primary tumor High tracer accumulation of ^99m^Tc-3P-RGD_2_ in right breast (**A.** left image and B left bottom two images, T/N=3.7) and left scapula (A: right image and B right upper two images) were demonstrated in one breast cancer patient with pulmonary nodule (**B.** left upper 2 images, T/N=1.08) in the left lung. The osteolytic bone metastasis in the left scapula displayed internal regional absence and slight elevation of ^99m^Tc-MDP uptake in the surrounding bone tissues (B: right bottom and C). A: ^99m^Tc-3P-RGD_2_ whole body scan; B: ^99m^Tc-3P-RGD_2_ SPECT-CT (the right bottom image is ^99m^Tc-MDP SPECT); **C.** ^99m^Tc-MDP whole body scan. (see arrows).

**Figure 5:**
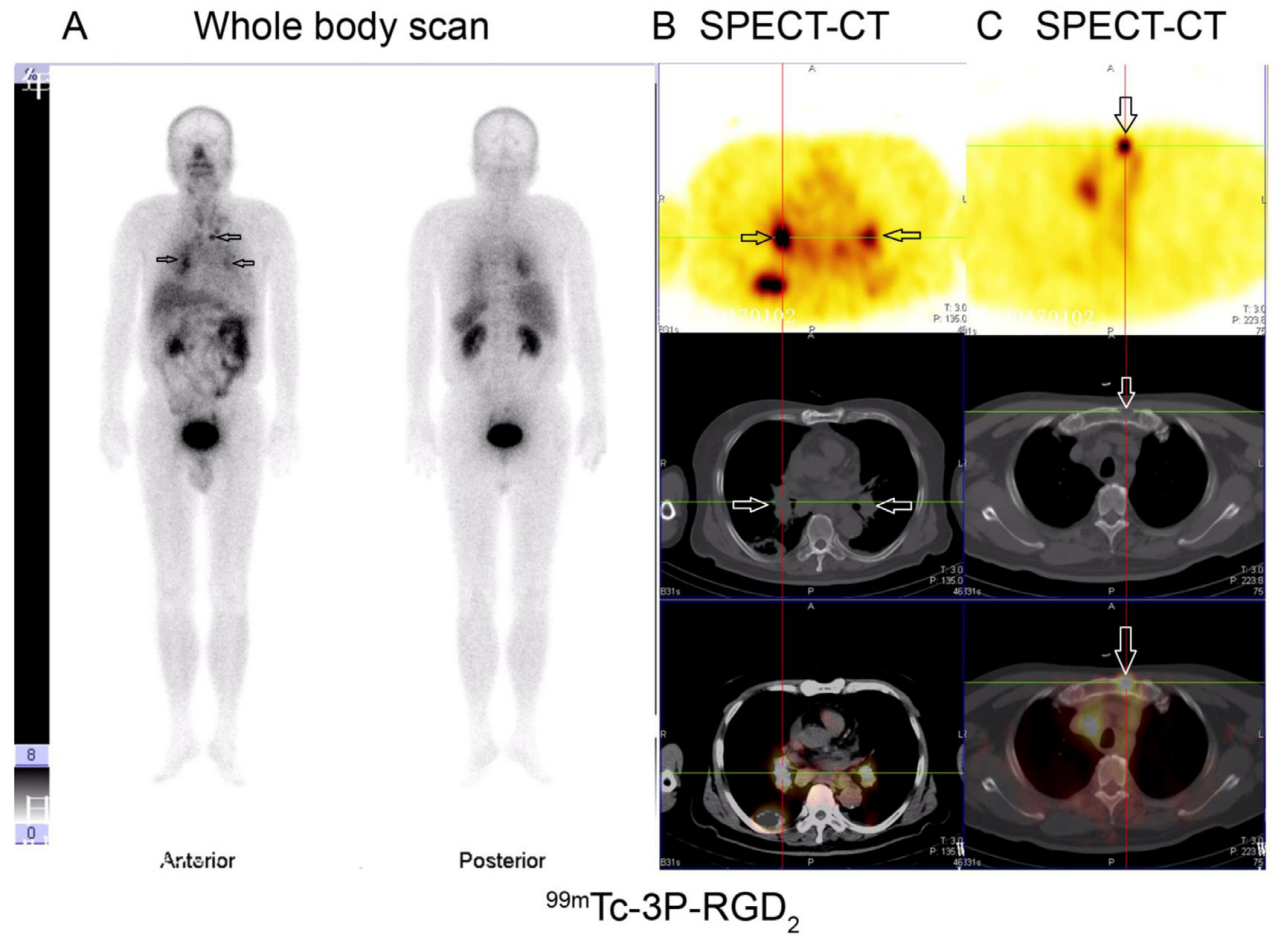
^99m^Tc-3P-RGD_2_ imaging of one patient with lung cancer and multi-lymph node metastases (mediastinal lymph node, hilar lymph node and presternal osteolytic) **A.** ^99m^Tc-3P-RGD_2_ whole body scan; **B.** and **C.** ^99m^Tc-3P-RGD_2_ SPECT-CT. (see arrows).

The score grade showed obvious change in both imaging modalities after SPECT-CT (Table 3). ^99m^Tc-3P-RGD_2_ imaging also displayed abnormal uptake in primary tumor and distant metastatic lesions (Figure 4 and 5, Table 1). Regional accumulation of ^99m^Tc-3P-RGD_2_ contributed to the detection of primary tumor in thyroid cancer (*n* = 3), breast cancer (*n* = 1), malignant chromaffin-cell tumor (*n* = 2), gastric cancer (*n* = 4), lymph node metastasis (Figure 5). On this basis, the clinical stage of the 69 patient was changed.

**Table 3:** Visual analysis of 131 osteolytic bone metastasis on ^99m^Tc-3P-RGD_2_ and ^99m^Tc-MDP WBS imaging and SPECT-CT

grade	WBS		WBS+SPECT-CT	
	^99m^**Tc-MDP**	^99m^**Tc-3P-RGD**_2_	^99m^**Tc-MDP**	^99m^**Tc-3P-RGD2**
0	54	20	27	10
1	15	29	27	32
2	28	54	39	61
3	34	28	38	28

### Detection value of ^99m^Tc-3P-RGD2 and ^99m^Tc-MDP imaging for osteolytic bone metastases

Semiquantification of tracer uptake in osteolytic bone mestastasis was expressed as T/N. A large variance (0.73-13.5) was noticed in the ^99m^Tc-3P-RGD2 in tracer uptake in osteolytic bone metastases. T/N on ^99m^Tc-3P-RGD_2_ WBS was significantly lower than that on SPECT-CT (4.93±2.20 vs. 6.80±3.19, t = 15.1, *p* < 0.01). The positive rate of ^99m^Tc-3P-RGD_2_ WBS showed significant increase compared to ^99m^Tc-MDP WBS based on T/N analysis (80.9% vs. 46.6%, *p* < 0.05, Supplemental Table 1). When combining with SPECT-CT, the detective sensitivity of ^99m^Tc-3P-RGD_2_ imaging increased to 96.2% (126/131). The osteolytic metastases demonstrated that lower or similar ^99m^Tc-3P-RGD_2_ uptake was more commonly seen in large size lesions with existence of predominant bone destruction, internal necrosis or/and surrounding cyclic hyperostosis/osteosclerosis. T/N of ^99m^Tc-3P-RGD_2_ in osteolytic metastatic lesions in lung adenocarcinoma patients showed no statitical difference from that in squamous-cell carcinoma (6.84±3.46 vs. 7.33±3.22, t = 0.39, *p* = 0.71). T/N of ^99m^Tc-3P-RGD_2_ in osteolytic metastases from primary lung cancer was significantly higher than that from thyroid cancer (7.05±3.01 vs. 4.11±2.67, *p* = 0.03).

## DISCUSSION

^99m^Tc-MDP is widely used to detect metastatic bone lesions via adhering to hydroxyapatite crystal and collagen in bone matrix. It is manifested as a “hot zone” in osteoblastic bone metastases even before changes of anatomical structure. ^99m^Tc-MDP WBS imaging is superior for the detection of osteoblastic metastases, however, its efficiency in the detection of osteolytic lesions is hampered due to overlaping from normal bone tissues. Osteolytic bone metastasis, characterized by the activation of osteoclasts and the resulting bone resorption, is visualized as a “cold area” (negative imaging) due to absence of ^99m^Tc-MDP uptake. However, osteolytic metastatic lesions, consisting of a great number of tumor cells and osteoclasts expressing high level of integrin αvβ3 and αvβ5 [23], are visualized as a “hot zone” (positive imaging) as it can bind to ^99m^Tc-3P-RGD_2_. This was the main mechanism of ^99m^Tc-3P-RGD_2_ imaging for the detection of osteolytic bone metastases with high T/N ratio. Our study showed that ^99m^Tc-3P-RGD_2_ WBS was more effective than ^99m^Tc-MDP WBS in the detection of osteolytic bone metastases, which may be related to the following aspects: Firstly, “hot area” was more easy to be visualized than “code area” on WBS. Secondly, distribution of ^99m^Tc-3P-RGD_2_ in normal bone structures was extremely low, which made the osteolytic bone metastases much easier to be detected with low background. Thirdly, overlaping of normal bone structures contributed to low grade for the lesions in vetebrea, sternum, sacrococcyx and pelvis, which finally induced misdiagnosis by ^99m^Tc-MDP WBS [24].

Clinically, SPECT-CT was performed in presence of abnormality or highly suspicious on WBS images. For the highly suspected patients, it is difficult for a patient to accept the whole body SPECT-CT each time during his first imaging or during regular follow-up. The detection of suspicious osteolytic bone metastases on WBS is of great importance as it serves as a gate for selective SPECT-CT imaging that will facilitate lesion detection, boundary delineation for semiquantitive analysis and accurate diagnosis [25, 26]. The detection value of osteolytic bone metastases increased by about 15% and several patients with score grade increased on both imaging modalities after combining with SPECT-CT. SPECT-CT is vital for ^99m^Tc-3P-RGD_2_ imaging due to absence of normal skeleton appearance for lesion localization. Furthermore, moderate-to-intense ^99m^Tc-3P-RGD_2_ accumulation in visceral organs might make the mild-uptake lesions undetectable in lower thoracic, upper lumber vertebra and sacrococcyx [27]. The diagnostic sensitivity of ^99m^Tc-3P-RGD_2_ imaging for osteolytic bone metastases was significantly higher than that of ^99m^Tc-MDP imaging in our study. Also, its sensitivity was slightly lower than ^18^F-Alfatide II PET-CT imaging [18]. This may be related to the higher spatial resolution of PET and the large database. Besides detection of osteolytic bone metastases, ^99m^Tc-3P-RGD_2_ imaging was also useful for the differential diagnosis of primary tumor and even its staging [14, 28]. In our study, primary lung cancer, thyroid cancer, malignant chromaffin-cell tumor and gastric cancer were highly positive on ^99m^Tc-3P-RGD_2_ imaging [29]. Thus, ^99m^Tc-3P-RGD_2_ imaging was superior to ^99m^Tc-MDP in the detection of osteolytic bone metastases, unknown primary tumor, distant metastases and clinical staging. T/N of ^99m^Tc-3P-RGD_2_ in osteolytic metastasis may be partially influenced by integrin αvβ3 expression level on primary tumor cells [30]. In future, further studies involving more cases of different malignant tumors are needed.

In metastatic bone tissues, ^99m^Tc-MDP imaging reflects bone metabolism and osteogenesis, while ^99m^Tc-3P-RGD_2_ imaging reflects the existence of integrin αvβ3–overexpressing tumor cells, osteoclast, angiogenesis [23, 31]. Osteolytic bone metastases usually demonstrated as increased accumulation of ^99m^Tc-3P-RGD_2_ imaging and low uptake of ^99m^Tc-MDP. However, “hot area” or “cold area” can be visualized on both imaging modalities at the same lesion in this study. The process of osteolytic bone metastases formation is triggered by the interaction among tumor cells, bone marrow environment and bone cells (vicious cycle) [32]. The tissue component changes during the “vicious cycle” until bone destruction and structure absence. “Hot area” on both imaging modalities is possibly due to increased activation of osteolysis and osteogenesis at early stage, while “cold area” is possibly associated with bone structure resorption, destruction, internal necrosis at late stage of osteolytic bone metastasis. The CT information on SPECT-CT is important to distinguish malignant osteolytic bone destruction from benign ones when they demonstrate as “cold area” on both ^99m^Tc-3P-RGD_2_ and ^99m^Tc-MDP imaging. Thus, these two imaging modalities are potential to play complementary roles in reflecting pathophysiological status of osteolytic lesions. In future, further studies are needed to develop targeted therapies for the management of osteolytic metastasis using 177Lu and 90Y labeled RGD peptide.

## CONCLUSIONS

Using integrin αvβ3 highly expressed in the main component of osteolytic bone metastases (e.g. tumor cells and osteoclasts) as a target, ^99m^Tc-3P-RGD_2_ imaging demonstrated mainly as “hot region” on whole body imaging with low background.

^99m^Tc-3P-RGD_2_ imaging showed significantly higher detective rate than ^99m^Tc-MDP imaging based on visual and semiquantitive analysis. Such technique is potentially applicable for the detection of some unknown primary tumors and distant metastases.

## SUPPLEMENTARY MATERIALS TABLE


