# Supplementary material for: Clinical study of 99mTc-3P-RGD2 peptide imaging in osteolytic bone metastasis
Source: Oncotarget. 2017 Apr 27;8(43):75587–96. doi: 10.18632/oncotarget.17486 (PMC5650448; doi:10.18632/oncotarget.17486)
Supplement: Supplementary file 1 [file oncotarget-08-75587-s001.pdf]

Clinical study of ^{99m}Tc-3P-RGD2 peptide imaging in osteolytic bone metastasis

Supplementary Material

Supplementary Table 1: Comparison of the diagnostic consistency of osteolytic bone metastases using ^{99m}Tc-3P-RGD$_2$ and ^{99m}Tc-MDP whole body scan imaging based on T/N

^{99m}Tc-MDP	^{99m}Tc-3P-RGD$_2$	
	+	-
+	60	1
-	46	24

$p<0.05$
